# Nasal Rhinomyiasis Caused by Cochliomyia hominivorax Complicated by a Parapharyngeal Abscess: A Case Report

**DOI:** 10.7759/cureus.100740

**Published:** 2026-01-04

**Authors:** Mauricio Megchún Hernández, Eleasib Alejandro Espinoza Pérez, Néstor Rodolfo M García Chong, Helen Ariadne Ralda Gómez, Diego Ivan Diaz-Haaz

**Affiliations:** 1 Paediatrics and Child Health, Hospital de Especialidades Pediátricas, Tuxtla Gutiérrez, MEX; 2 Surgery, Faculty of Human Medicine "Dr. Manuel Velasco Suárez" Campus II, Universidad Autónoma de Chiapas, Tuxtla Gutiérrez, MEX; 3 Biostatistics, Faculty of Human Medicine "Dr. Manuel Velasco Suárez" Campus II, Universidad Autónoma de Chiapas, Tuxtla Gutiérrez, MEX; 4 Paediatrics, Hospital de Especialidades Pediátricas, Tuxtla Gutiérrez, MEX; 5 Orthopaedics, Faculty of Human Medicine "Dr. Manuel Velasco Suárez" Campus II, Universidad Autónoma de Chiapas, Tuxtla Gutiérrez, MEX

**Keywords:** chiapas, cochliomyia hominivorax, ivermectin, nasopharyngeal myiasis, parapharyngeal abscess

## Abstract

Nasopharyngeal myiasis is a rare parasitic disease caused by *Cochliomyia hominivorax* larvae that affects the nasal cavity and paranasal sinuses. A 12-year-old adolescent presented with a lesion caused by a fly entering the left nostril, characterised by the emergence of larvae. Sinus tomography documented inflammatory changes consistent with ethmoid-maxillary sinusitis in the left nasal cavity and parapharyngeal abscess. Treatment included ivermectin for myiasis, clindamycin and cefotaxime for the abscess, and functional endoscopic sinus surgery (FESS) for cleaning and debridement. The patient's clinical course was favourable after multidisciplinary treatment, and she was discharged after improvement.

## Introduction

Myiasis is the infestation of dipteran larvae in the skin, mucous membranes, and organs of living vertebrates. The term comes from the Greek mya (fly) and iasis (disease) [1]. There are several known vectors that cause this disease, including *Cochliomyia hominivorax *(blue-green fly) from the American continent, *Dermatobia hominis *from Central and South America, and *Cordylobia anthropophaga* from the sub-Saharan region [2,3]. In Mexico, this disease was eradicated in 1991, but reappeared in November 2024 in a foreign-born bovine in the state of Chiapas. On 17 April 2025, the first human case was reported in the municipality of Acacoyagua, Chiapas [4]. Clinically, myiasis has been subdivided into: sanguinivorous myiasis, dermal and subdermal myiasis, nasopharyngeal myiasis, intestinal myiasis, and urogenital myiasis [5]. Nasopharyngeal myiasis is a rare parasitic disease that causes damage to the nasal cavity and paranasal sinuses. The characteristic clinical picture includes pain, nasal bleeding, foul-smelling discharge, and swelling of the lower eyelids, nose, and upper lip [6]. It has been documented that the larvae migrate to surrounding structures such as the eyes and skull cavity, causing severe complications. Flies are attracted to natural body openings and open wounds, where they lay their eggs, and the larvae feed on the host's living or dead tissue [7].

## Case presentation

The patient's mother gave her consent for the relevant findings to be presented and published. The following is a description of a rare case of myiasis, with informed consent.

**Figure 1:**
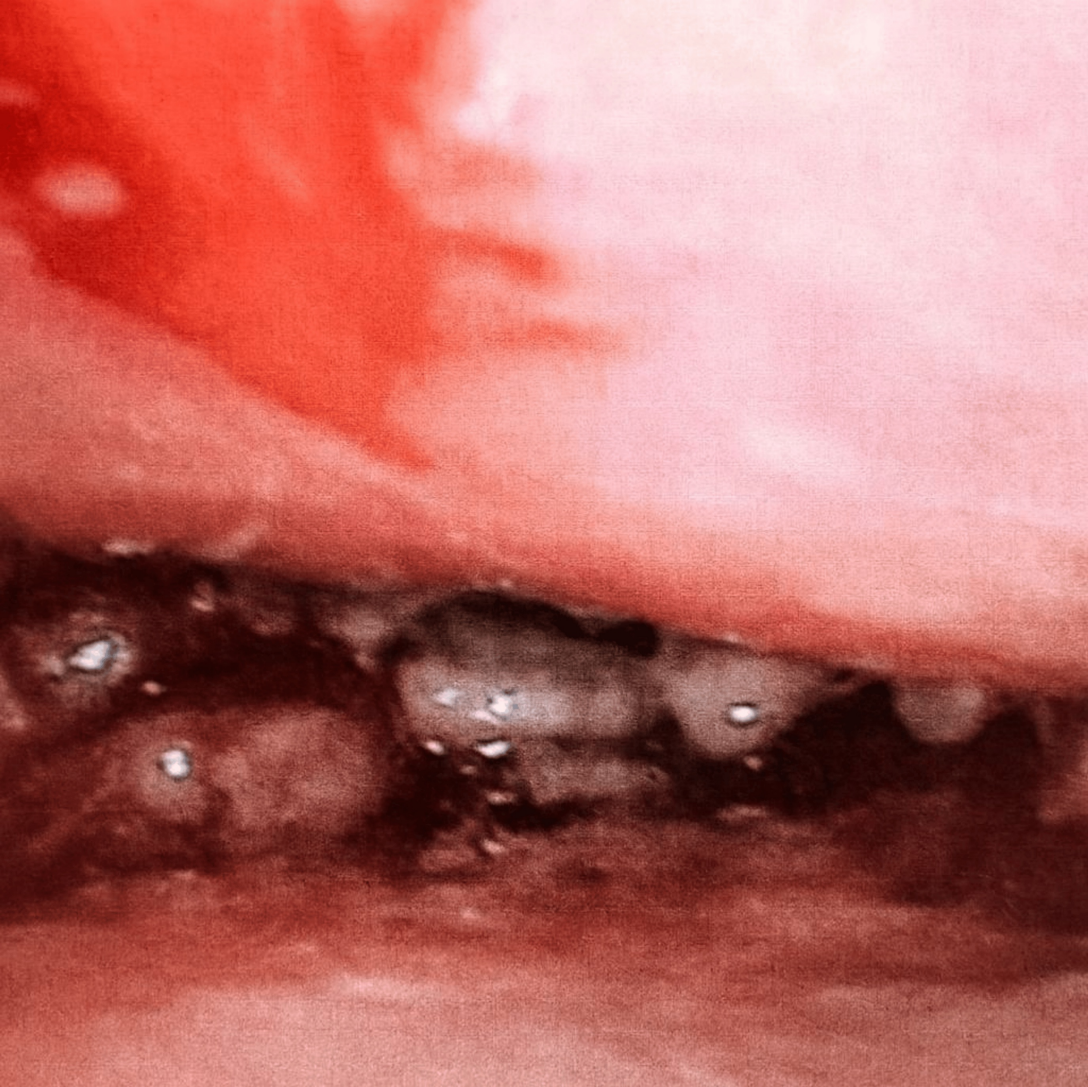
Left nasopharyngeal cavity showing the presence of larvae measuring approximately 4 mm in length.

We present the case of a 12-year-old female adolescent patient, previously healthy, originally from and resident in the municipality of Ocosingo, Chiapas, México, who speaks the indigenous Tzeltal language. The condition began on 11 August 2025 at primary school, when she reported that a fly had entered her left nostril, causing nasal itching and hyaline rhinorrhoea, remaining asymptomatic for several days. Subsequently, she presented with pain in the left maxilla radiating to the ipsilateral nostril, accompanied by a cough with greenish expectoration and the presence of larvae. For this reason, she was taken to the hospital in her community, where some larvae were removed. A few days later, the patient reported pain in the left ear canal and headache, for which she was referred to the Paediatric Speciality Hospital. Upon admission, she was assessed by the Epidemiology, Otolaryngology, and Paediatric Infectious Diseases departments. On physical examination, the patient was conscious and alert, with stable vital signs (see Table 1). In addition, larvae approximately 4 mm in length were observed in the left nostril, bilateral hyaline rhinorrhoea, and a parapharyngeal abscess was evident in the oral cavity (Figure 1). The larvae were preserved in 70% ethyl alcohol and placed in a screw-top plastic jar, which was stored in the hospital laboratory. They were subsequently sent to the state public health laboratory, where the taxonomic diagnosis of *C. hominivorax* was confirmed. On the other hand, laboratory tests and wound cultures were performed on the patient, reporting positivity for *Pseudomonas aeruginosa* (see Table 1).

**Table 1:** Laboratory tests and vital signs.

Parameter	Result	Units	Reference values
Blood cytometry			
Haemoglobin	11.4	g/dL	12.9-16.3
Haematocrit	34.7	%	39.5-49.1
Mean corpuscular volume	87.4	fL	80.9-96.6
White blood cells	8.67	x10^3^/mm^3^	3.60-10.70
Lymphocytes	21	%	18-57
Monocytes	8	%	3-9
Eosinophils	2	%	0-5
Segmented	69	%	32-71
Platelets	389	x10^3^/µL	167-431
Acute phase reactants			
C-reactive protein	7.53	mg/dL	0.000-0.500
Procalcitonin	0.07	ng/mL	0-0.50
Erythrocyte sedimentation rate	89	mm/h	3-13
Immunoglobulin			
Immunoglobulin E (IgE)	130	UI/mL	100-150
Cultivate			
Wound culture	Positive	-	Pseudomonas aeruginosa
Vitals signs			
Heart rate	78	beats/min	60-100
Respiratory frequency	18	breaths/min	12-20
Temperature	37	°C	36.5-37.5
Blood pressure	110/70	mmHg	90-120/60-80
Oxygen saturation	98	%	>94

**Figure 2:**
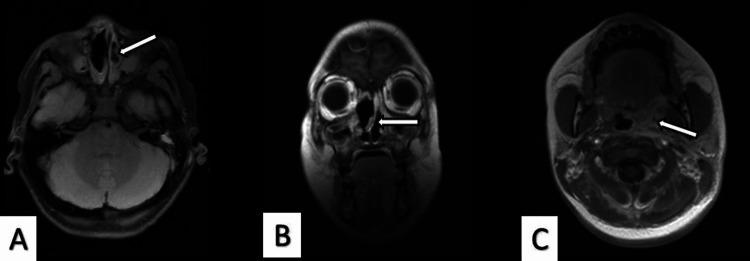
Cranial CT scan findings. (A) Axial image showing ethmoid-maxillary sinusitis in the left nasal cavity (arrow indicating the left maxillary sinus). (B) Coronal image showing mucosal thickening in both maxillary sinuses, bilateral anterior osteomeatal complex obstruction, and septal deviation (arrow indicating the nasal septum). (C) Axial image showing a hypodense collection in the left parapharyngeal region consistent with an abscess (arrow indicating the left parapharyngeal region).

Computed tomography (CT) of the paranasal sinuses revealed agenesis of the frontal sinuses, images of greater attenuation at the level of the ethmoid cells, and thickening of the mucosa in both maxillary sinuses. Inflammatory changes consistent with ethmoid-maxillary sinusitis in the left nasal cavity, bilateral anterior osteomeatal complex obstruction, septal deviation, left palatine tonsil hypertrophy, and a hypodense collection in the left parapharyngeal region were also documented (Figure 2).

Medical treatment was initiated empirically with clindamycin and cefotaxime for the left parapharyngeal abscess, supplemented with ivermectin for myiasis. After obtaining the wound culture results, the antibiotic regimen was adjusted. Cefotaxime was discontinued, and cefepime was added for 10 days. The otolaryngology service performed endoscopic surgical treatment using functional endoscopic sinus surgery (FESS), which consisted of surgical cleaning and debridement of the nasal cavity. The anterior ethmoid bone and the ethmoid bulla on the right side were excised, revealing purulent material in the right maxillary sinus. On the left side, the natural ostium was enlarged, but no purulent material was found in that area. During the patient's hospital stay, a deep abscess of the left parapharyngeal neck was observed, which was treated subsequently with surgical drainage and completion of the antimicrobial regimen. The patient's clinical evolution was favourable, and she was discharged due to improvement.

## Discussion

*C. hominivorax*, known as the blue-green fly, is a species native to the American continent and one of the main causative agents of myiasis. The female lays her eggs in clusters of between 12 and 400, usually on mucous membranes or open wounds in animals and humans. The larvae emerge a few hours later, favoured by warm and humid environmental conditions, and then begin to feed on the host's living tissue, causing progressive and potentially severe damage [8]. Nasopharyngeal myiasis is often considered an accidental nasal infestation, most commonly observed in rural areas where people tend to have direct contact with animals [9]. The present case is consistent with the above description, as it involves a resident of a rural area who accidentally acquired the infection with similar clinical characteristics. On the other hand, the patient presented major complications reported by the CT scan, including a parapharyngeal abscess, septal deviation, and ethmoid-maxillary sinusitis. In contrast, studies have been reported in which only mild effects were observed, such as erythematous mucosa, permeable nasal passages, and no deviation of the nasal septum [10]. The treatment of myiasis involves manual and/or surgical removal in cases of migratory forms [11]. In addition, irritants or asphyxiants have been used to expel the larvae from the affected site, including creolin, turpentine oil, sulphuric ether, varnish, eucalyptus oil, turpentine oil, and lidocaine spray [12]. Ivermectin has also been used as an antiparasitic agent sensitive to dipteran larvae such as *C. hominivorax* [13,14]. In our case, the patient was treated with manual and surgical endoscopic extraction via FESS, as well as with ivermectin, clindamycin, and cefotaxime for the parapharyngeal abscess.

## Conclusions

Nasopharyngeal myiasis is a rare form of this parasitic disease, whose occurrence has been associated mainly with rural areas and contexts of socioeconomic vulnerability. Although previously under control, in recent years it has re-emerged as a public health problem in Mexico that requires immediate notification. Delayed diagnosis increases the risk of severe complications, making it a priority for health services at all levels of care to strengthen early identification capabilities and ensure timely and appropriate management of cases.
